# Severe Covid‐19 and acute pulmonary hypertension: 24‐month follow‐up regarding mortality and relationship to initial echocardiographic findings and biomarkers

**DOI:** 10.1111/aas.14168

**Published:** 2022-11-28

**Authors:** Joakim Norderfeldt, Andreas Liliequist, Staffan Eksborg, Claes Frostell, Maria J. Eriksson, Christofer Adding, Per Agvald, Per‐Arne Lönnqvist

**Affiliations:** ^1^ Department of Clinical Physiology Karolinska University Hospital Stockholm Sweden; ^2^ Department of Molecular Medicine and Surgery Karolinska Institutet Stockholm Sweden; ^3^ Section of Thoracic Anaesthesia and Intensive Care, Perioperative Medicine and Intensive Care Karolinska University Hospital Stockholm Sweden; ^4^ Pediatric Perioperative Medicine and Intensive Care and Division of Paediatrics, Astrid Lindgren Children's Hospital Karolinska University Hospital Solna Stockholm Sweden; ^5^ Childhood Cancer Research Unit, Department of Women's and Children's Health Karolinska Institutet Stockholm Sweden; ^6^ Department of Anaesthesia and Intensive Care Karolinska Institutet at Danderyd Hospital Stockholm Sweden; ^7^ Section of Urology, Department of Molecular Medicine and Surgery Karolinska Institutet Stockholm Sweden; ^8^ Section of Pharmacology, Department of Physiology and Pharmacology Karolinska Institutet Stockholm Sweden; ^9^ Section of Anaesthesiology and Intensive Care, Department of Physiology and Pharmacology Karolinska Institutet Stockholm Sweden

**Keywords:** 24‐month follow up, Covid‐19, echocardiography, NTproBNP, outcome, pulmonary hypertension, acute, troponin‐T

## Abstract

**Introduction:**

Critically ill Covid‐19 patients are likely to develop the sequence of acute pulmonary hypertension (aPH), right ventricular strain, and eventually right ventricular failure due to currently known pathophysiology (endothelial inflammation plus thrombo‐embolism) that promotes increased pulmonary vascular resistance and pulmonary artery pressure. Furthermore, an in‐hospital trans‐thoracic echocardiography (TTE) diagnosis of aPH is associated with a substantially increased risk of early mortality. The aim of this retrospective observational follow‐up study was to explore the mortality during the 1–24‐month period following the TTE diagnosis of aPH in the intensive care unit (ICU).

**Methods:**

A previously reported cohort of 67 ICU‐treated Covid‐19 patients underwent an electronic medical chart‐based follow‐up 24 months after the ICU TTE. Apart from the influence of aPH versus non‐aPH on mortality, several TTE parameters were analyzed by the Kaplan–Meier survival plot technique (K‐M). The influence of biomarkers for heart failure (NTproBNP) and myocardial injury (Troponin‐T), taken at the time of the ICU TTE investigation, was analyzed using receiver‐operator characteristics curve (ROC) analysis.

**Results:**

The overall mortality at the 24‐month follow‐up was 61.5% and 12.8% in group aPH and group non‐aPH, respectively. An increased relative mortality risk continued to be present in aPH patients (14.3%) compared to non‐aPH patients (5.6%) during the 1–24‐month period. The easily determined parameter of a tricuspid valve regurgitation, allowing a measurement of a systolic pulmonary artery pressure (regardless of magnitude), was associated with a similar K‐M outcome as the generally accepted diagnostic criteria for aPH (systolic pulmonary artery pressure >35 mmHg). The biomarker values of NTproBNP and Troponin‐T at the time of the TTE did not result in any clinically useful ROC analysis data.

**Conclusion:**

The mortality risk was increased up to 24 months after the initial examination in ICU‐treated Covid‐19 patients with a TTE diagnosis of aPH, compared to non‐aPH patients. Certain individual TTE parameters were able to discriminate 24‐month risk of morality.

**Editorial Comment**

In this study, longer follow‐up is presented for patients with echocardiographic evidence of pulmonary hypertensive during admission for severe Covid‐19, a condition that was previously shown to have a higher 1‐month mortality. For those patients surviving past 1 month, the survival in the next 23 months of follow‐up did not differ between patients with and without pulmonary hypertension. Several echocardiographic markers of pulmonary hypertension had an association with overall mortality during the entire 2‐year follow‐up period.

## 1 INTRODUCTION

Research performed during the first wave of Covid‐19 did reveal several different pathophysiologic processes associated with the disease. Among these pathophysiologies, two different processes that could influence the pulmonary circulation were identified. First, Covid‐19 was found to cause a generalized inflammation of the endothelium, endothelitis, that promotes vasoconstriction of the small lung vessels due to reduced production of vasodilators (e.g., nitric oxide and prostacyclin).^1^ Second, the endothelitis combined with a generalized coagulopathy promotes micro‐ and macro‐embolization of the lungs.^2^ Furthermore, the pulmonary endothelitis of the microcirculation in the lung was found to result even in in‐situ thrombosis of the small pulmonary vessels.^3^ Taken together this sets the stage for acute pulmonary hypertension (aPH). Several studies subsequently reported that the occurrence of aPH in hospitalized Covid‐19 patients was found to be high^4^, ^5^, ^6^, ^7^, ^8^ and that the presence of aPH, with or without simultaneous right ventricular dysfunction, was associated with substantially increased mortality.^4^, ^5^, ^6^, ^7^, ^8^ In our previous study on this topic, we found an incidence of aPH, based on echocardiographic criteria, of 39% in Covid‐19 patients admitted to intensive care (ICU) and that early mortality (21 days) was increased more than 6‐fold compared to patients without signs of aPH.^4^

In previous follow‐up reports on hospitalized Covid‐19 patients,^9^, ^10^ the issue of aPH has not been reported in any detail. Against the results of our previous study, we hypothesized that mortality may continue to be elevated after 1 month in patients with Covid‐19 and aPH. Thus, the primary aim of the present study was to perform a retrospective observational 24‐month follow‐up in Covid‐19 patients that received ICU care during the first wave of the pandemic, with a special focus on the 1–24‐month period. This to evaluate if aPH continues to affect mortality during this time period (early [0–1‐month] mortality already reported).^4^ Secondary aims were to analyze whether various trans‐thoracic echocardiographic (TTE) parameters as well as certain biomarkers (e.g., NTproBNP and Troponin‐T), taken at the time of diagnosis of aPH in the ICU, were related to increased long‐term mortality (0–24‐month time period).

## 2 METHODS

For detail of the initial study, please see Appendix. In brief, 67 patients with severe Covid‐19, treated at the various ICUs at the Karolinska University Hospital, during the time period of April 10, 2020–May 17, 2020 were included in the original study. All Covid‐19 patients admitted to ICU care during the study period did by default undergo a TTE, to better understand the hemodynamics of this new disease. All TTE were performed by a certified echocardiographer (author J.N.) and the diagnosis of aPH was based on international consensus guidelines.^11^, ^12^, ^13^, ^14^ At the time of the TTE, blood samples were taken and analyzed with regards to the biomarkers NTproBNP (proxy for heart failure) and Troponin‐T (proxy for myocardial injury). Twenty‐six patients were diagnosed with aPH (group aPH) whereas 41 did not meet aPH criteria (group non‐aPH).

In the present follow‐up study, the 1‐month survivors in group aPH (*n* = 14) and group non‐aPH (*n* = 36) of the initial study were included (relevant patient characteristics presented in Table 1). For the secondary outcomes all patients of the initial study were included (for relevant data, please see Appendix).

**TABLE 1:** Patient data for patients still alive 30 days after the trans‐thoracic echocardiography (present study population)

	aPH (*n* = 14)	non‐aPH (*n* = 36)
Age (years); median (range)	57 (37–69)	58 (34–74)
Sex (male/female)	13/1	34/2
Weight (kg); median (range)	89 (70–145)	85 (66–116)
sPAP (mmHg); median (range)	48 (42–67)	30 (22–35)^a^
mTR	14	7
PAAT (<90 ms)	5 [2]	8 [2]
IVCnrc	5 [1]	6 [1]
TAPSE (<17 mm)	1	7

*Note*: [] = missing data.

Abbreviations: aPH, acute pulmonary hypertension; IVCnrc, no respiratory changes in the inferior vena cava; mTR, measurable tricuspid valve regurgitation; PAAT, pulmonary artery acceleration time; sPAP, systolic pulmonary artery pressure.

^a^ Relates to the 7 patients in group non‐aPH having a mTR.

The issue of bias was addressed by the fact that ICU‐admitted Covid‐19 patients during the study period underwent TTE by default and that all TTE were performed by the same certified echocardiographer.

### 2.1 24‐month mortality follow‐up

Following Ethics Committee approval of an amendment to the original study (EPM Dnr 2022‐00126‐02, Chairperson Gunilla Robertsson. Informed consent was, as with the initial study, waived by the Ethics committee), the patients' electronic medical charts were reviewed 24 months after the initial TTE in the ICU. This electronic medical chart is cross‐linked to the Swedish Death registry and is up‐dated daily. Mortality was the only parameter investigated in this study. Thus, no further chart review was performed since a substantial number of patients had been transferred back to their home region for further care following discharge from our hospital. Mortality was assessed up to 24‐ months after the diagnostic TTE.

The primary aim of the study was to compare mortality during the *time period 1–24 months* in Covid‐19 patients classified as having TTE‐classified aPH versus non‐aPH during their initial ICU stay.

Regarding the secondary aims (see below), *the entire 0–24‐month period* was evaluated.

### 2.2 TTE parameters

The TTE parameters examined in the initial study are shown in the Appendix. A special interest was focused on one parameter that can easily be determined by a less trained echocardiographer; the presence of a tricuspid valve regurgitation that allowed a measurement of tricuspid transvalvular systolic pressure gradient. Thus, the tricuspid regurgitation was analyzed as being present and measurable or not and no focus was paid to the actual maximal regurgitant velocity. This analysis resulted in patients with a measurable tricuspid valve regurgitation (group mTR) versus patients without this finding (group non‐mTR).

Thus, in the present follow‐up study, we focused on whether the following five TTE parameters did relate to 24‐month survival: estimated sPAP >35 mmHg (definition of aPH), mTR, no respiratory changes in Inferior Vena Cava diameter (IVCnrc), Pulmonary Artery Acceleration Time (PAAT) (normal: >90 ms),^13^ and Tricuspid Annular Plane Systolic Excursion (TAPSE) (normal: >17 mm).^12^

### 2.3 Statistics

#### 2.3.1 TTE parameters

Primary and secondary aim parameters were subjected to conventional Kaplan–Meier survival curve statistics.

In parameters showing a statistically significant K‐M plot analysis regarding mortality at 24‐months (Figure 2), the following data were also generated as per standard procedure: specificity, sensitivity, negative predictive value (NPV), positive predictive value (PPV), positive likelihood ratio (PLR), and negative likelihood ratio (NLR).

##### Biomarkers

The values of NTproBNP and Troponin‐T were subjected to receiver‐operator characteristics curve (ROC) analyses.

*p*‐values <.05 were considered as statistically significant.

## 3 RESULTS

No patient was lost to follow‐up. There were only isolated missing data with regards to the five chosen TTE parameters (Table 1).

### 3.1 Primary aim

**FIGURE 1:**
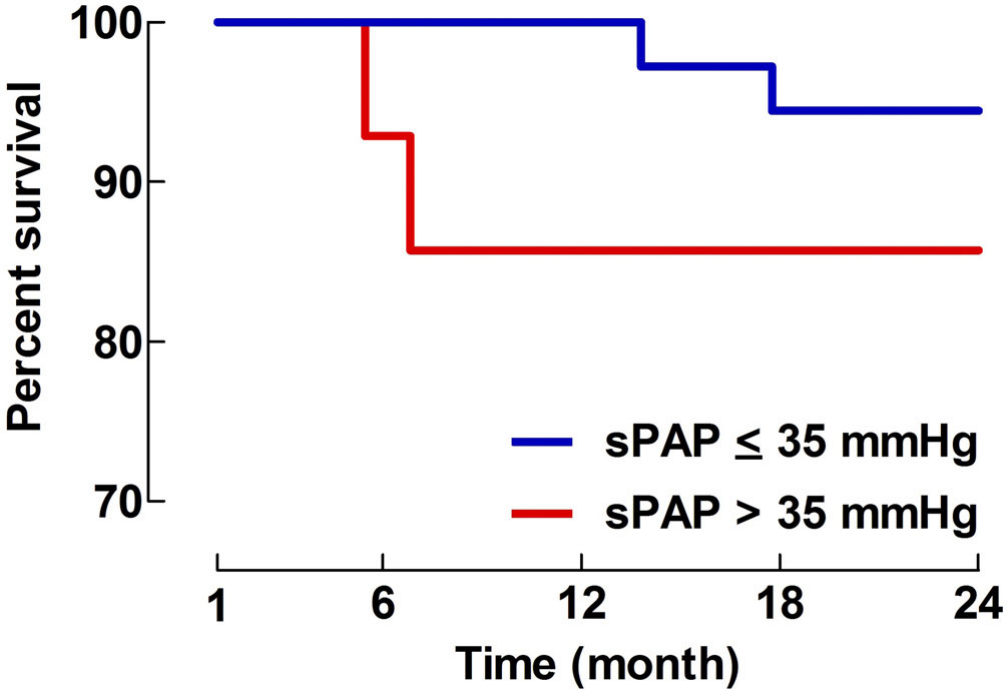
Kaplan–Meier (K‐M) survival plot for systolic pulmonary artery pressure (sPAP) >35 mmHg (=aPH) and sPAP ≤35 mmHg (=non‐aPH) for the time period 1–24‐months. The number of patients at the time points 1‐ and 24‐months were 14 versus 36 and 12 versus 34 in groups sPAP <35 mmHg and sPAP ≤35 mmHg, respectively. *p*‐value based on K‐M statistics.

At 1‐month post‐TTE 14 (of 26) and 36 (of 41) patients were still alive in group aPH and non‐aPH, respectively. An additional 2 patients died during the 1–24‐month period in group aPH, the corresponding number in group non‐aPH being 2 patients. Thus, the fraction of patients dying in group aPH and non‐aPH during this time period was 2/14 (14.3%; 95% CI: 1.8–42.8) and 2/36 (5.6%; 95% CI: 0.7–18.7), *p* = .2806. Mortality for the aPH and non‐aPH groups during the 1–24‐month period is displayed as K‐M survival plots in Figure 1.

### 3.2 Secondary aims

#### 3.2.1 TTE parameters

**FIGURE 2:**
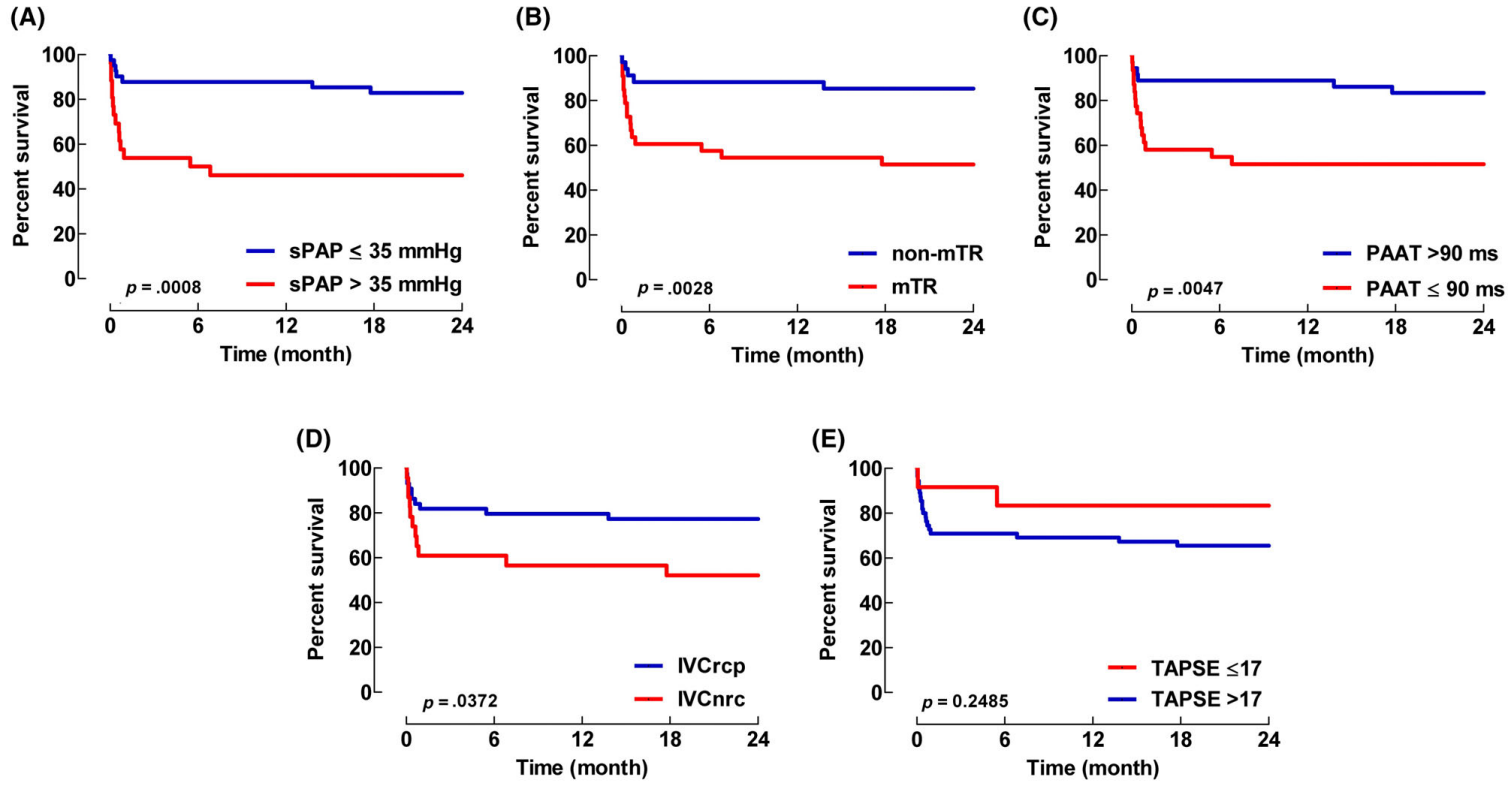
K‐M survival plots for echocardiographic parameters for time period 0–24‐months. The number of patients at the time points 0, 1‐ and 24‐months were 26 versus 41, 14 versus 36, 12 versus 34, in groups sPAP >35 mmHg and sPAP ≤35 mmHg, respectively. Systolic pulmonary artery pressure (sPAP), measurable tricuspid valve regurgitation (mTR), pulmonary artery acceleration time (PAAT), no respiratory changes in the inferior vena cava (IVCnrc), and tricuspid annular plane systolic excursion (TAPSE), are shown in panels (A–E), respectively. IVCrcp, respiratory changes observed in inferior vena cava. *p*‐values based on K‐M statistics.

K‐M survival plots for the five investigated TTE parameters for the time period 0–24‐months are shown in Figure 2A–E.

Of these parameters, sPAP, mTR, PAAT, and IVCnrc resulted in significant *p*‐values (.0008, .0028, .0047 and .0372, respectively) at the end of the 0–24‐month observation period. No significant difference was seen for TAPSE.

In Table 2 the specificity, sensitivity, NPV, PPV, PLR, and NLR are presented for the four parameters (sPAP, mTR, PAAT, and IVCnrc) associated with significantly reduced survival regarding the 0–24‐month observation period.

**TABLE 2:** Specificity, sensitivity, negative predictive value (NPV), positive predictive value (PPV), positive likelihood ratio (PLR), and negative likelihood ratio (NLR) for the four statistically significant TTE parameters, as per the Kaplan–Meier (KM) plot analyses regarding 0–24‐month mortality

	sPAP	mTR	PAAT	IVCnrc
Specificity	0.829	0.853	0.545	0.524
95% CI	0.679–0.928	0.689–0.950	0.3221–0.756	0.298–0.743
Sensitivity	0.538	0.485	0.167	0.227
95% CI	0.334–0.734	0.308–0.665	0.0637–0.328	0.115–0.378
NPV	0.739	0.630	0.286	0.244
PPV	0.667	0.762	0.310	0.500
PLR	3.15	3.3	0.37	0.48
NLR	0.56	0.6	1.53	1.48

Abbreviations: 95% CI, 95% confidence interval; IVCnrc, no respiratory changes in the inferior vena cava; mTR, measurable tricuspid valve regurgitation; PAAT, pulmonary artery acceleration time; sPAP, systolic pulmonary artery pressure.

#### 3.2.2 Biomarkers

**FIGURE 3:**
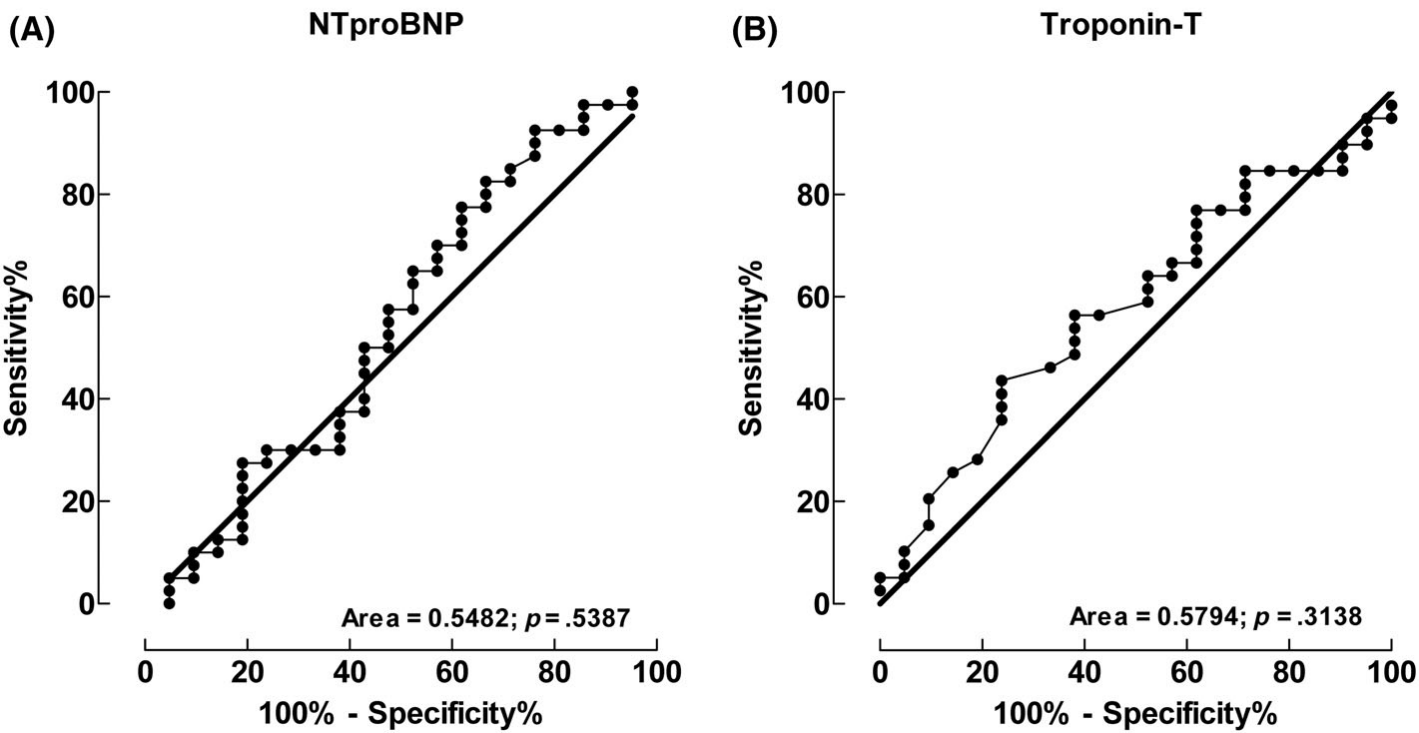
Receiver‐operator characteristics curve analysis for biomarkers regarding 0–24‐month mortality. NTproBNP and Troponin‐T are shown in panels (A, B), respectively.

Plasma levels of NTproBNP and Troponin‐T taken at the time of the TEE did not yield any significance with regards to mortality at the 24‐month time point. The ROC curves for NTproBNP and Troponin‐T are displayed in Figure 3.

## 4 DISCUSSION

The main finding of the present 24‐month follow‐up study was that ICU‐treated Covid‐19 patients from pandemic wave 1, with an echocardiographic diagnosis of aPH, continued to show a relative increase in mortality during the 1–24‐month period following the diagnosis, compared to patients not diagnosed with aPH. Certain individual TTE parameters were able to discriminate 24‐month risk of morality.

The early lack of interest in the issue of aPH is illustrated by the fact that a comprehensive review of the circulatory implications of Covid‐19, published during the first pandemic wave, did not even mention aPH.^15^ However, following case series of patients dying from Cor Pulmonale,^16^ reports on the incidence of aPH in hospitalized Covid‐19 were subsequently published.^4^, ^5^, ^6^, ^7^, ^8^ These studies showed an incidence of echocardiographically diagnosed aPH of approximately 40% depending on the setting (normal ward or ICU).^4^, ^5^, ^6^, ^7^, ^8^ Furthermore, patients diagnosed with aPH were found to suffer substantially increased death rates during the early clinical course (early mortality 37%–46%).^4^, ^5^

Due to this striking difference in mortality, in the future studies on severe Covid‐19, we find it essential to stratify the study population into patients displaying aPH on a TTE versus patients lacking this finding in order not to draw the wrong conclusions. One can in fact argue that the presence of TTE diagnosed aPH in this patient population represents a biomarker for a high risk of mortality.

Since aPH is a condition caused by a serious underlying pathology, combined with that even a shorter period with overload and distension of the heart might cause persistent damage (i.e., increased risk for arrythmia), we found it reasonable to assume that the mortality risk would remain increased into the intermediate future.

### 4.1 Mortality

In our previous study, we showed that aPH patients showed significantly higher early mortality compared to non‐aPH patients.^4^ As hypothesized, the fraction of patients diagnosed with aPH during their ICU stay displayed a higher mortality rate relative to non‐aPH patients during the timeframe of 1–24 months after the diagnosis, albeit this relative difference (14.3% vs. 5.6%) did not achieve statistical significance. The reason for the lack of significance may either be due to a true lack of difference between the two groups or, maybe more likely, to the small number of patients remaining for analysis during the 1–24 months period. Thus, the remaining population was most likely underpowered to find any statistically significant difference regarding mortality.

Based on the grim mortality rate in patients that are diagnosed with aPH, it seems that considerable clinical effort should be directed at reducing aPH to protect the right ventricle. However, the obvious choice of administering inhaled nitric oxide (iNO) has not been shown to be generally successful in patients with severe Covid‐19.^17^ This could potentially be due to iNO being unable to reach all relevant parts of the pulmonary circulation due to the specific nature of the Covid‐19 pneumonia. The use of a selective intravenous pulmonary vasodilator might potentially be more effective and such a trial is currently in the recruitment phase (ClinicalTrials.gov Identfier: NCT04885491; “A study to evaluate the Efficacy, Tolerability and Safety of PDNO infusion in Covid‐19 patients with aPH”).

To possibly prevent later mortality, we agree with a recent Editorial comment^18^ that ICU‐treated Covid‐19 aPH patients should benefit from close follow‐up, under the auspice of a cardiologist or physician with a similar competency, for at least 1 year after the initial hospitalization. This may apply to any ICU patient, regardless of etiology, that is diagnosed with aPH during their ICU stay. However, whether our Covid‐19 findings regarding aPH can be generalized to include also other ICU pathologies remains to be investigated in future clinical trials.

### 4.2 TTE parameters

Out of the parameters investigated, we conclude that, apart from the accepted definition of aPH (sPAP >35 mmHg), only three (mTR, PAAT, and IVCnrc) resulted in a significant 0–24‐month K‐M plot analysis, indicating that abnormal values are associated with earlier mortality.

The analysis of specificity, sensitivity, NPV, PPV, PLR, and NLR with regards to mortality (Table 2) did show that sPAP and mTR were associated with substantially better values than those for PAAT and IVCnrc. Thus, the sPAP and mTR appear as the preferred parameters in this context.

Our finding that mTR produce a K‐M survival plot that is in close agreement with the much more skill demanding procedure of achieving a trustworthy measurement of sPAP >35 mmHg, and is interesting from the clinical perspective. However, this obviously needs to be validated in further ICU aPH studies.

Of note is that TAPSE below the cut‐off value of 17 mm, somewhat surprisingly, did not relate to an increased mortally risk. This is contrary to a previous report by d'Alto et al.^6^ who found a reduced value of TAPSE as a marker for bad outcome. However, Huang et al. have also found TAPSE to be of unclear value in Covid‐19 ICU patients.^19^, ^20^ Hopefully further studies will be able to delineate the usefulness of TAPSE in seriously ill Covid‐19 patients.

### 4.3 Biomarkers

When investigated at a group level, NTproBNP and Troponin‐T were both clearly higher in group aPH versus non‐aPH at the time of the TTE diagnosis.^4^ However, when analyzed at an individual level (ROC curve analysis), no clinically useful cut‐off values could be identified for these two proxy parameters with regards to 24‐month mortality.

### 4.4 Study limitations

As with the original study, the major limitation of the present study is that the diagnosis of aPH is based on TTE findings and not on the gold standard of invasive pulmonary artery catheterization. However, during the very demanding circumstances in the ICU during the first wave of Covid‐19, pulmonary artery catheterization could not be regularly accomplished. Thus, our findings need to be verified by further studies in this context using invasive monitoring as a complement to TTE. A second limitation is the relatively small sample size that remined for analysis after the 30‐day post‐TTE period. A larger sample size may well have produced more reliable data. Thus, further studies, including larger cohorts of patients, need to be performed to confirm or refute our non‐statistically significant finding of a higher mortality rate in aPH versus non‐aPH patients during the time period 1–24 months. The fact that all echocardiographic examinations were performed by the same operator (author J.N.), who is a certified echocardiographer, obviously interferes with generalization of our results to TTE examinations performed by less skilled colleagues. Further, to use mTR instead of sPAP >35 mmHg will be associated with a slight risk of over‐diagnosing aPH since several patients with a mTR did show estimated pulmonary artery pressure values below 35 mmHg. Thus, the presence of a mTR should not be viewed solely as a proxy for aPH but more as a sign of any disease process that may cause dilatation of the right ventricle, making the tricuspid valve incompetent (e.g., overdistention due to aPH or dilatation due to direct cardiomyopathy caused by Covid‐19 myocarditis).

In conclusion, a higher mortality rate was present in ICU‐treated Covid‐19 patients diagnosed with aPH (according to the established TTE definition of sPAP >35 mmHg) compared to non‐aPH patients, not only during the early phase of the disease, but also during the 1–24‐month period after the diagnosis of aPH. Certain individual initial TTE parameters were found able to discriminate 24‐month risk of morality.

## AUTHOR CONTRIBUTION

JN: performance of TTE. Data retreval. Data analysis. Manuscrpit writing. AL: Data retrevial. Biomarker analyses. Data retreval. SE: Statistical analyses. MJE: Echocardiographic interpretations and analyses. CF: Data retreval. Data analyses. Manuscript writing. CA: Data retreval. Manuscript review. PA: Dta retreval. Manuscript review. PAL: Primary investigator, involved in all aspects of the study.

## FUNDING INFORMATION

No funding was received for this study.
